# Review essay: *politics, power, and universal health care* by Stuart Altman and David Schachtman

**DOI:** 10.1186/2045-4015-1-23

**Published:** 2012-05-23

**Authors:** David Chinitz

**Affiliations:** 1 Braun School of Public Health, Faculty of Medicine, Hebrew University, Jerusalem, Israel

## 

In public discourse regarding health policy in Israel, one often hears that “we are on the road to America”. The concern underlying this cry is that the public–private mix in Israel, especially in terms of finance, is slowly wending its way towards the U.S. situation in which more than half of the national health expenditure comes from private sources. This is, of course, a somewhat skewed contention, as it disregards the fact that all Israeli residents are guaranteed a broad package of health entitlements, while in the U.S., fifteen percent of the population is uninsured. The attraction of simplistic, one-dimensional comparisons is great, especially in the heat of social debate. This tendency toward simplification exists not only in Israel, but also in the U.S. and other countries as well. It is good that parallel to this, a less strident and more considered discussion sometimes takes place among health policy analysts, providing understanding that, hopefully, takes its place in the maelstrom of the policy process.

In the U.S. context, Stuart Altman and David Schactman have chosen to play this edifying role in *Power, Politics, and Universal Health Care*. In this fascinating and informative chronicle of U.S. health policy over the course of almost a century, Altman relies on his unique combination of academic grounding and continuous presence in the trenches of the battle for universal health insurance in America. Altman is a Brandeis University professor, and has served in many administrations and been involved as a task member in every health reform effort since 1970. Few, if any, observers, especially when teamed up with a knowledgeable and savvy co-author like Schactman, can give the reader both inside stories of how the policy process works, together with an analytical framework to mold the raw history into policy-relevant learning.

It is an old adage that policy, like sausage, is a phenomenon regarding which it is perhaps better not to know the actual production process. Altman convincingly contradicts this dictum, demonstrating how the intricate workings of policy making, including the vagaries of individual behavior and the timing of unexpected, and seemingly unrelated, events are essential elements missed by the bird’s eye view of national health policy studies. One might describe the book as “health policy made human”.

Nothing captures this better than Altman’s Law: “Most every major health care constituent group favors universal coverage and health care reform, but if it deviates from their preferred approach they would rather remain with the status quo.” This predicament will sound readily familiar to Israelis, who ask themselves why it is that – on the diplomatic front – most would like to have peace and not to “rule over another people”, but prefer the status quo if the proposed solution is not their cup of tea. And, yet, how is it that Altman’s law applies to health policy in America, but not in Israel, where not only has health coverage been nearly universal since before the creation of the state, but major changes have taken place even over the objections of major constituent groups?

Part of the answer lies in Altman’s description of the various efforts, beginning with the Nixon presidency through to the current administration, to adopt universal health care. All of these efforts are characterized by what Jim Morone has called the “democratic wish” that public policy should not be made by a distant government but, rather, by the people [1]. This desire, entrenched in the political culture of America, paradoxically, seeks for policy to be democratic, but also depoliticized, or at least devoid of government dominance. This leads to what others have called the “technocratic wish”, namely, that policy, especially in a field like medical care, which is considered a science, should be based on objective, professional criteria. In the U.S., these wishes readily combine with the preference for the free market – in which consumers exercise choice in consuming health care, the latter having been defined as some kind of homogenous well understood product much like tomatoes or widgets – as opposed to “big government”.

Thus, as described by Altman, Richard Nixon was interested in promoting universal health care, but wanted to ground this in what he perceived as market-based solutions that would not be perceived by his Republican constituency as big government. Seeking to head off competition from Edward Kennedy, a leading democratic contender for the Presidency and the leading proponent of national health insurance, Nixon enacted legislation that required employers to offer their employees the option to obtain health coverage from health maintenance organizations (HMO). Nixon, familiar with Kaiser Permanente that had its roots in his home state of California, perceived that this type of pre-paid group practice would, in the context of market competition, provide cheaper and higher quality care, thus enabling more Americans to obtain coverage. But pre-paid group practice, sounding a bit too “socialized” and at odds with the mainstream medical profession, based on solo, fee for service, practice, had to be morphed into HMOs. The HMOs that formed to meet the requirements of Nixon’s plan were mainly open panel, non-group practice, and exercised relatively little management over their providers. Ultimately, not only did Nixon fail to enact his Comprehensive Health Insurance Plan, in which Altman had a major role in crafting, also he did not need to worry about Kennedy who became embroiled in a personal scandal that derailed his presidential ambitions at the time.

This legacy of efforts to enact national health care reform based on technical, market based, ostensibly de-politicized mechanisms, congruent with Altman’s Law, is repeated in the Clinton plan to base reform on “managed competition”. As described by Altman, the technocratic, secretive policy task force led by Hillary Clinton created too much suspicion and, despite its purported intent to be market based, was portrayed by the insurance industry as a recipe for government limitations on freedom of choice for patients and providers in the health system.

It is in the Obama administration that, according to Altman, politics asserts itself in a way that cannot be suppressed in the health reform debate. Perhaps learning from the Clinton experience, President Obama did not propose his own plan, but waited for a combination of proposals from the Congress that he could mold into legislation that would, and eventually did, pass. Coming at the same time as the collapse and bailout of the financial system, Obama could not “hide” the need for regulations that would not only reform unpopular practices of the insurance industry, but would also lead to cost savings that would balance the cost of expanding coverage. For the first time since the enactment of Medicare and Medicaid, the federal government dealt first with expanding coverage, and put off cost containment to the future. As Altman points out, it remains to be seen how much of cost containment will actually be implemented and to what degree it will succeed.

When presented to Israeli students of health policy, this history of U.S. health policy is nearly incomprehensible. How, they ask, can a political culture operate on the assumption that health care should be organized without a strong role for government? How can the American system, to which many Israelis, rightly or wrongly, look for examples in sound and accountable fiscal policy, seek to expand coverage without simultaneously dealing with cost containment?

These Israelis perhaps forget that for forty-six years various efforts to nationalize health insurance encountered severe resistance in the form of a preference for the pluralistic system of private not-for-profit sickness funds, and the “politicization” inherent in the trilateral linkage among the Histadrut, the Labor Party, and Kupat Cholim Clalit. It was, in part, this politicization of health that created financial instability in the system, so that reform was readily linked to cost containment. Since enactment of NHI in Israel, the politics of health have dealt head on with the tension between the desire for more services and the need to control the budget. Many of the cost containment provisions in the Obama Affordable Care Act, such as cost effectiveness research and integrated accountable care organizations, are already deployed in Israel, underpinning the system of universal coverage. The politics of entitlements are institutionalized in mechanisms such as the committee to update the basket of services.

Observers from other western health systems, looking at Israelis looking at the U.S. story as told by Altman, will exclaim that, in all of their systems, governments first made coverage universal and then began to deal with cost containment. For reasons of history and politics, Israel has become extremely explicit about cost containment, perhaps more so than any other country. Other Western systems are increasingly overlaying their long history of universal coverage and global budgets with explicit rules about practice and entitlements. Ironically, the U.S. is the source of many of the tools, such as managed care, DRGs, risk-adjusted capitation, and cost effectiveness research, that are being deployed elsewhere within national health systems. This demonstrates the fact that such technical tools of health system management and regulation are not stand alone solutions, but need to be enveloped in political institutions that can overcome Altman’s law.

The question is whether the Obama Affordable Care Act can meet those challenges and, if it does, whether it will succeed in bringing about universal coverage and, eventually, cost containment of the ever growing U.S. health expenditure. While the book by Altman and Schachtman does not purport to make a prediction on this, it is a history that provides deeper understanding of how the U.S. system has evolved. This is important not only for U.S. readers, for whom this book makes the debate about their own health system more accessible, but also for those from other countries who sometimes tend to dismiss the U.S. as, at best, an example of how not to organize a national health system. By looking at the U.S. story, readers from other countries can also better understand how their own systems developed, and the importance of political institutions that make it possible to overcome Altman’s law. Israelis can be reminded that the technical tools of quality assurance and cost containment are necessary, but not sufficient tools for managing the health system that also requires mechanisms, such as the committee to update the basket of services, to frame the politics of health. Other Western systems with universal coverage can look at the U.S. and Israel and perhaps bask in the knowledge that their own combination of universal coverage and budgetary controls have been in place for a long time, but they might also consider the tensions highlighted by stories such Altman’s and the degree to which they will need to refine their institutions in order to keep what they have.

## Competing interests

The authors declare that they have no competing interests.

## Author information

David Chinitz is a Professor of Health Policy and Management at the School of Public Health, Hebrew University-Hadassah, Jerusalem. He currently serves as President of the International Society for Priority Setting in Health Care.
